# Patterns and correlates of cannabis use by cumulative lifetime violence severity as target and/or perpetrator in a community sample of eastern Canadian men

**DOI:** 10.1186/s42238-020-00021-5

**Published:** 2020-03-23

**Authors:** Sue O’Donnell, Kelly Scott-Storey, Judith Wuest, Jeannie Malcolm, Petrea Taylor, Charlene D. Vincent

**Affiliations:** 1 Faculty of Nursing, University of New Brunswick, P.O. Box 4400, Fredericton, New Brunswick E3B 5A3 Canada; 2 Faculty of Nursing, University of New Brunswick, Moncton, Canada

**Keywords:** Cannabis, Lifetime cumulative violence, Men, Gender, Health, Health promotion, Substance use

## Abstract

**Background:**

Recent Canadian legalization of cannabis for non-medical use underscores the need to understand patterns and correlates of cannabis use among men who may be more likely than women to become problematic cannabis users. Evidence supporting an association between substance use and violence is accumulating. Current knowledge of relationships among patterns of cannabis use, violence, gender and health is limited by dichotomous measurement of cannabis use and a focus on individual types of violence rather than lifetime cumulative violence.

**Methods:**

We collected online survey data between April 2016 and Septermber 2017 from a community convenience sample of 589 Eastern Canadian men ages 19 to 65 years and explored how socio-demographic characteristics, gender, and health varied by past-year patterns of cannabis use (i.e., *daily, sometimes, never*) in the total sample and by higher and lower cumulative lifetime violence severity (CLVS) measured by a 64-item CLVS scale score (1 to 4).

**Results:**

Overall prevalence of cannabis use was 46.6% and differed significantly between lower (38.1%) and higher (55.3%) CLVS groups (*χ*^*2*^ (1) = 17.42, *p* = .000). *Daily* cannabis use was more likely in the higher (25.1%) than the lower group (11.9%, *χ*^*2*^ (2) = 31.53, *p* < .001). In the total sample, *daily* use was significantly associated with being single, less education, lower income, some gender norms, health problems, and use of other substances. Significant associations were found for *sometimes* cannabis use with age group 19 to 24 years, being single, some gender norms, and hazardous and binge drinking. *Never* use was associated with being married, more education, higher income, being older, not using other substances, and not having mental health problems. Associations between cannabis use patterns and many variables were found in both CLVS groups but effect sizes were frequently larger in the higher group.

**Conclusions:**

These results add substantively to knowledge of relationships among lifetime cumulative violence, patterns of cannabis use, gender, socio-demographic indicators and health problems and may inform theoretical models for future testing. Additionally, findings provide critical information for the design of health promotion strategies targeted towards those most at risk in the current climate of cannabis legalization.

## Background

The legalization of cannabis in Canada in October 2018 has heightened the importance of understanding patterns and outcomes of cannabis use among Canadians as a basis for strategic health promotion. Trends in past-year cannabis use found in national surveys conducted from 2004 to 2015 have shown a gradual increase in consumption for Canadians ages 25 to 64 years, a finding that supports studying patterns of cannabis use across the lifespan (Rotermann and Macdonald 2018). Use of cannabis by Canadian men requires closer study because, in comparison to women, men have been found to be 1.6 times more likely to try cannabis and 2.5 times more likely to exhibit problematic cannabis use (Bonner et al. 2017). Although understanding patterns of use according to sub-groups is essential for research and health-related interventions, many reports do not differentiate findings for men and women. Comparison of findings among Canadian population surveys is also difficult because the time period (e.g., ever, past 12 months, past 3 months) for indicating whether cannabis has been used varies among surveys, and frequency of use (e.g., daily, weekly, monthly) in that period is not always collected (Hango and LaRochelle-Côté 2018). Further, across studies, definitions for *problematic* or *risky* use can range in frequency from *monthly* to *daily*; a recent systematic review suggested that *weekly use* or greater was commonly considered risky with *daily or almost daily* being problematic (Casajuana et al. 2016).

Substance use is associated with violence, a global public health problem that is a major source of morbidity and mortality for men (Haegerich and Hall 2011). However, much research supporting the relationship between cannabis use and individual types or forms of violence is limited by neglect of potential confounders including gender and exposure to other violence experiences (Ostrowsky 2011; Scott-Storey 2011). Despite these limitations, knowledge supporting a relationship between cannabis use and individual types or forms of violence as a target and perpetrator is accumulating (Ostrowsky 2011). For example, physical and/or sexual childhood maltreatment and peer drug use predicted heavy cannabis use in adolescent males (Dubowitz et al. 2016), past year male criminal victimization (physical assault, sexual assault, robbery) was significantly associated with almost daily cannabis use in the past month (Hango and LaRochelle-Côté 2018), and intimate partner violence perpetration was associated with cannabis use by men arrested for domestic violence (Shorey et al. 2018). Because multiple experiences of violence are common across the lifespan (Scott-Storey 2011), consideration of how patterns of cannabis use are affected by not only individual types of violence but also cumulative exposure to violence across the lifespan is essential. There are no such studies of which we are aware, possibly because no measure of lifetime cumulative violence existed. To address this limitation we developed the Cumulative Lifetime Violence Severity (CLVS) scale (Scott-Storey et al. 2018).

The *Men’s Violence, Gender and Health Study (MVGHS)* was designed to explore differences in men’s health according to lifetime cumulative violence (Scott-Storey et al. 2018). We conducted the *MVGHS* in New Brunswick (NB), Canada from April 2016 to September 2017, prior to cannabis legalization for non-medical use, in a community sample of 589 men and measured CLVS and cannabis use in the past 12 months as well as a range of other socio-demographic and health indicators. In this exploratory analysis, we used these data to examine patterns and correlates of cannabis use and to illuminate how lifetime cumulative violence influences these associations. Specifically, we asked: 1) how the descriptive profile (CLVS, socio-demographic characteristics, gender norms [beliefs about what it means to be a man], health status, and substance use) of the 589 men varied by pattern of cannabis use, and 2) how the descriptive profile by pattern of cannabis use for men with higher perceived CLVS compared to that of men with lower CLVS. Knowledge of these bivariate relationships is critical for developing theoretical models to test the relationships among patterns of cannabis use, health, socio-demographic indicators, gender and CLVS.

## Methods

After receiving approval from the Research Ethics Board of the affiliated university and informed consent from each participant, we collected data for the *MVGHS* using an online survey with a community convenience sample of individuals who self-identified as men, were ages 19 to 65 years, and lived in NB (Scott-Storey et al. 2018). Exposure to violence in one’s lifetime was not an inclusion criterion for taking part. Recruitment was primarily through online classified advertisements as well as through posters and supporters who shared information about the study in workplaces and community settings. Men first contacted the study coordinator by email or telephone, and if interested were sent the letter of information and an online link to determine eligibility. Those who provided online consent were directed to the online survey. Men who completed the survey received an honorarium of 20 Canadian dollars (CAD). The survey included scales and self-report questions of socio-demographic characteristics, gender, lifetime cumulative violence, health, and substance use. Patterns of cannabis use were measured with a single item asking how often cannabis had been used in the past year (never, once or twice, monthly, weekly, or daily/almost daily). In this analysis, categories of use were collapsed to 3: *never*, *sometimes* (once or twice, monthly, or weekly) or *daily* (daily or almost daily).

The CLVS scale measures severity as frequency and distress of physical, psychological, and sexual violence experiences from childhood (under 18 years of age) through adulthood, as target and/or perpetrator, and in the context of gender, families, intimate relationships, schools, communities, and workplaces (Scott-Storey et al. 2018). Each of the 64 items were rated on 4-point scales (1 to 4) for both frequency (*never* to *often*) and distress (*not at all* to *very distressing*). Responses to frequency and distress for each item were summed and averaged for a possible severity score from 1 to 4. Severity scores were summed and averaged for a total CLVS score (range 1 to 4; α = .94.) with higher CLVS indicated by a score greater than 1.32, the median scale score for the sample (Scott-Storey et al. 2018).

Masculine gender role was assessed by the 46-item Conformity to Masculine Norms Inventory-46 (CMNI-46), a multi-dimensional scale which measures conformity to dominant masculine gender norms (Parent and Moradi 2009). The CMNI-46 has 9 sub-scales (range 0 to 3) which include Winning (α = .84), Emotional Control (α = .91), Risk-taking (α = .84), Violence (α = .85), Primacy of Work (α = ..77), Playboy (α = .80), Self-reliance (α = .86), Power over Women (α = .78) and Homosexual Self-presentation (α = .89). Possible depression was measured with a score greater than 15 (range 0 to 60) on the 20-item Center for Epidemiologic Studies-Depression Revised (CESD-R) scale, α = .95 (Eaton et al. 2004). Possible Posttraumatic Stress Disorder (PTSD) was appraised by a score greater than 34 (range 17 to 85) on the 17-item PTSD Checklist, Civilian Version (PCL-C), α = .95 (Blanchard et al. 1996). Possible moderate to severe anxiety was measured by a score greater than 9 (range 0 to 21) on the Generalized Anxiety Disorder 7-item (GAD-7) scale, α = .93 (Spitzer et al. 2006). Low disability chronic pain was defined by a Chronic Pain Grade (CPG) of 0, I or II and high disability chronic pain by a CPG of III or IV, derived from the CPG Scale (Von Korff et al. 1992). Possible hazardous drinking was assessed with a score greater than 3 (range 0 to 12) using the 3-item 5-point Audit Alcohol Consumption screen (AUDIT-C), provided the total score did not come only from the frequency of drinking item (Bradley et al. 2007).

### Analysis

Using SPSS© Version 25 software, we conducted descriptive analyses to identify significant differences by pattern of cannabis use using the Chi-square Test for frequencies in categorical data, determining significantly different cell values by standardized residuals greater than ± 1.96. The magnitude of the relationship (effect size) among variables was determined by interpreting Cramer’s V according to Chi-square degrees of freedom (Kim 2017). For continuous data, we used analysis of variance (ANOVA) using Welch’s *F* when the assumption of homogeneity of variance was not met. Games-Howell post-hoc testing was used to determine significant differences in group mean scores (Field 2013) with Eta-squared used to estimate the effect size (Polit 2010). These analyses were conducted for the total NB sample and for the higher and lower CLVS groups.

## Results

### Cumulative lifetime violence severity

Of the 589 men in this sample, 97.5% of the participants reported lifetime exposure to violence with 15.3% as a target only, 0.5% as perpetrator only and 81.7% as both. The mean CLVS score for the total sample was 1.40 (range 1.00 to 2.73). The lower CLVS group (*n* = 294) had a mean CLVS score of 1.16 (range 1.00 to 1.32) and the higher CLVS group (*n* = 295) had a mean CLVS score of 1.65 (range 1.32 to 2.73).

### Cannabis use

The two CLVS groups differed significantly with the higher CLVS group reporting greater prevalence of cannabis use in the past 12 months, *χ*^*2*^ (1) = 17.42, *p* = .000, V = .172, effect size (ES) = small. Prevalence was 46.6% (*n* = 275) in the total sample, 38.1% (*n* = 112) in the lower CLVS group and 55.3% (*n* = 163) in the higher group. In the total sample, 21% (*n* = 124) had used cannabis *daily*, 25.6% (*n* = 151) *sometimes*, and 53.3% (*n* = 314) *never* used in the past year. CLVS scores differed significantly by cannabis use patterns in the total sample (*F* (2, 586) = 19.18, *p* = .000, *η*^2^ = .061, medium ES). Mean CLVS scores for *daily* users (*n* = 124; μ = 1.55, SD = 0.35) were significantly higher than those for *sometimes* users (*n* = 151; μ = 1.40, SD = 0.32) and never users (*n* = 314; μ = 1.35, SD = 0.29).

Patterns of use were significantly different between the two CLVS groups (*χ*^*2*^ (2) = 31.53, *p* < .001, V = .231, ES = medium), with those in the higher group more likely to use cannabis *daily* and less likely to use *never.* In the lower CLVS group, 11.9% (*n* = 35) had used cannabis *daily*, 26.2% (*n* = 77) *sometimes*, and 61.9% (*n* = 182) *never* used. In the higher CLVS group, 30.2% (*n* = 89) had used *daily*, 25.1% (*n* = 74) *sometimes*, and 44.7% (*n* = 32) *never* used. No significant differences were found among CLVS mean scores by cannabis use patterns for the lower CLVS group (*F* (2, 291) = 0.93, *p* = .394) with mean CLVS scores for *daily* users being (*n* = 35; μ = 1.17, SD = 0.09), for *sometimes* users (*n* = 77; μ = 1.16, SD = 0.10) and never users (*n* = 182; μ = 1.15, SD = 0.09). Similarly, no differences were found among cannabis use patterns in the higher CLVS group (*F* (2, 292) = 2.55, *p* = .080) with mean CLVS scores for *daily* users being (*n* = 89; μ = 1.70, SD = 0.30), for *sometimes* users (*n* = 74; μ = 1.65, SD = 0.32) and never users (*n* = 132; μ = 1.61, SD = 0.26).

### Socio-demographic indicators

For the total sample, significant differences among patterns of cannabis use were found for all socio-demographic indicators (see Table 1). *Daily* cannabis use was significantly associated (ES = medium) with being single, divorced or separated, having a high school diploma or less education, living in a large city, being unemployed, earning less than $25,000 CAD annually, and having difficulty living on current income*. Sometimes* cannabis use was significantly associated (ES = medium) with being ages 19 to 24 years and being single, divorced or separated. *Never* use of cannabis was significantly associated with being married or cohabiting, having a college diploma or university degree, being employed, earning more than $50,000 CAD annually, not having difficulty living on income, and being ages 45 to 64.

**Table 1:** Patterns of cannabis use in past year by socio-demographic indicators for the total sample (*N* = 589)

	Patterns of Cannabis Use (*N* = 589)			Chi-square Test
	Never (53.3%, *n =* 314) ^a^	Sometimes (once or twice, monthly or weekly) (25.6%, *n* = 151) ^a^	Daily or Almost Daily (21%, *n* = 124) ^a^	
Age Groups: *n* (%)				
-19 to 24	33 (10.5)^b^	39 (25.8)^b^	25 (20.2)	*χ*^*2*^ (4) = 27.36, *p* = .000, V = .152
-25 to 44	164 (52.2)	81 (53.2)	69 (55.6)	
-45 to 65	117 (37.3) ^b^	31 (20.5)^b^	30 (24.2)	
Marital Status: *n* (%)		(*n* = 149)	(*n* = 123*)*	
-Married or Living with Partner	227 (72.3) ^b^	70 (47.0) ^b^	53 (43.1) ^b^	*χ*^*2*^ (2) = 44.83, *p* = .000, V = .277
-Single, Divorced or Separated	87 (27.7) ^b^	79 (53.0) ^b^	70 (56.9) ^b^	
Highest Level of Education: *n* (%)			(*n* = 123)	
-High School Diploma or less	58 (18.5) ^b^	36 (23.8)	48 (39.0) ^b^	*χ*^*2*^ (4) = 37.71, *p* = .000, V = .179
-Some Post-Secondary Education	74 (23.6)	49 (32.5)	41 (33.3)	
-College or University Degree/Diploma	182 (58.0) ^b^	66 (43.7)	34 (27.6) ^b^	
Community Size: *n* (%)		(*n* = 150)		
-Rural (< 1000)	43 (13.7)	19 (12.7)	13 (10.5)	*χ*^*2*^ (6) = 20.11, *p* = .000, V = .131
-Small town (1000 to 29,999)	81 (25.8)	29 (19.3)	14 (11.3) ^b^	
-Medium city (30,000 to 99,999)	166 (52.9)	88 (58.7)	75 (60.5)	
-Large city (> 100,000)	24 (7.6)	14 (9.3)	22 (17.7) ^b^	
Currently Employed: *n* (%)				
-Yes	240 (76.4)	111 (73.5)	59 (47.6) ^b^	*χ*^*2*^ (2) = 36.44, *p* = .000, V = .249
-No	74 (23.6) ^b^	40 (26.5)	65 (52.4) ^b^	
Total Personal Income Past Year: *n* (%)	(*n* = 313)	(*n* = 150)		
< $25,000	98 (31.3) ^b^	72 (48.0)	78 (62.9) ^b^	*χ*^*2*^ (4) = 58.14, *p* = .000, V = .223
$25,000 to $49,999	65 (20.8)	53 (23.3)	31 (25.0)	
= > $50,000	150 (47.9) ^b^	43 (28.7)	15 (12.1) ^b^	
Difficulty Living on Income: *n* (%)				
-Not at all or somewhat difficult	233 (74.4) ^b^	89 (59.3)	45 (36.6) ^b^	*χ*^*2*^ (2) = 55.00, *p* = .000, V = .306
-Difficult to extremely difficult	80 (25.9) ^b^	78 (40.7)	78 (63.4) ^b^	

^a^ Unless otherwise specified

^b^ Standardized Residual > ± 1.96

In the higher CLVS group, patterns of cannabis use differed significantly by all indicators similar to that of the total sample. However, in the higher group, the magnitude of association was large between being single and *daily* and *sometimes* cannabis use, and between having an income less than $25,000 CAD annually and *daily* use. In the lower CLVS group, patterns of cannabis use did not differ significantly by community size or employment, but daily use was significantly associated (ES = medium) with being single, divorced or widowed, having a high school education or less, and having difficulty living on one’s income. No significant associations with sociodemographic indicators were found for *sometimes* or *never* use in the lower CLVS group (Table 2).

**Table 2:** Patterns of cannabis use in past year by socio-demographic indicators by lower and higher CLVS^a^ scale score groups

	Lower CLVS (*n* = 294) Patterns of Cannabis Use				Higher CLVS (*n* = 295) Patterns of Cannabis Use			
	Never (61.9%, *n =* 182)^b^	Sometimes (26.2%, *n* = 77)^b^	Daily (11.9%, *n* = 35) ^b^	Chi- square	Never (44.7%, *n =* 132) ^b^	Sometimes (25.1%, *n* = 74) ^b^	Daily (30.2%, *n* = 89) ^b^	Chi- square
Age Groups: *n* (%)								
-19 to 24	23 (12.6)	18 (23.4)	9 (25.7)	*χ*^*2*^ (4) = 13.57, *p* = .009, V = .152	10 (7.6)^c^	21 (28.4)^c^	16 (18.0)	*χ*^*2*^ (4) = 22.06, *p* = .000, V = .193
-25 to 44	99 (54.4)	42 (54.5)	23 (65.7)		65 (49.2)	39 (52.7)	46 (51.7)	
-45 to 65	60 (33.0)	17 (22.1)	3 (8.6)^c^		57 (43.2)^c^	14 (18.9)^c^	27 (30.3)	
Marital Status: *n* (%)		(*n* = 76)				(*n* = 73)	(*n* = 88)	
-Married or Living with Partner	124 (68.1)	41 (53.9)	14 (40.0)	*χ*^*2*^ (2) = 11.98, *p* = .003, V = .202	103 (78.0)^c^	29 (39.7) ^c^	39 (44.3)	*χ*^*2*^ (2) = 38.59, *p* = .000, V = .363
-Single, Divorced, or Separated	58 (31.9)	35 (46.1)	21 (60.0)^c^		29 (22.0) ^c^	44 (60.3) ^c^	49 (55.7) ^c^	
Highest Level of Education: *n* (%)			(*n* = 34)					
-High School Diploma or less	26 (14.3)	16 (20.8)	11 (32.4)^c^	*χ*^*2*^ (4) = 10.80,*p* = .029, V = .136	32 (24.2)	20 (27.0)	37 (41.6) ^c^	*χ*^*2*^ (4) = 22.55, *p* = .000, V = .196
-Some Post-Secondary Education	47 (25.8)	22 (28.6)	12 (35.3)		27 (20.5)	27 (36.5)	29 (32.6)	
-College or University Degree/Diploma	109 (59.9)	39 (50.6)	11 (32.4) ^c^		73 (55.3) ^c^	27 (36.5	23 (25.8) ^c^	
Community Size: *n* (%)		(*n* = 76)						
-Rural (< 1000)	22 (12.1)	11 (14.5)	3 (8.6)	*χ*^*2*^ (6) = 5.25, *p* = .512, V = .095	21 (15.9)	8 (10.8)	10 (11.2)	*χ*^*2*^ (6) = 19.22, *p* = .004, V = .180
-Small town (1000 to 29,999)	43 (23.6)	12 (15.8)	5 (14.3)		38 (28.8)	17 (23.0)	9 (10.1) ^c^	
-Medium city (30,000 to 99,999)	101 (55.5)	45 (59.2)	21 (60.0)		65 (49.2)	43 (58.1)	54 (60.7)	
-Large city (> 100,000)	16 (8.8)	8 (10.5)	6 (17.1)		8 (6.1)	6 (8.1)	16 (18.0) ^c^	
Currently Employed: *n* (%)								
-Yes	145 (79.7)	60 (77.9)	22 (69.2)	*χ*^*2*^ (2) = 4.75, *p* = .093, V = .127	95 (72.0)	51 (68.9)	37 (41.6) ^c^	*χ*^*2*^ (2) = 22.84, *p* = .000, V = .278
-No	37 (20.3)	17 (22.1)	13 (37.1)		37 (28.0)	23 (31.1)	52 (58.4) ^c^	
Total Personal Income Past Year: *n* (%)	(*n* = 181)	(*n* = 76)						
< $25,000	56 (30.9)	28 (36.8)	19 (54.3)	*χ*^*2*^ (4) = 14.87,*p* = .005, V = .160	42 (31.8) ^c^	44 (59.5)	59 (66.3) ^c^	*χ*^*2*^ (4) = 42.02, *p* = .000, V = .267
$25,000 to $49,999	39 (21.5)	21 (27.6)	11 (31.4)		26 (19.7)	14 (18.9)	20 (22.5)	
= > $50,000	86 (47.5)	27 (35.5)	5 (14.3)^c^		64 (48.5) ^c^	16 (21.6)	10 (11.2) ^c^	
Difficulty Living on Income: *n* (%)								
-Not at all or somewhat difficult	143 (79.0)	52 (68.4)	17 (48.6)	*χ*^*2*^ (2) = 14.56, *p* = .001, V = .223	90 (68.2) ^c^	37 (50.0)	28 (31.8) ^c^	*χ*^*2*^ (2) = 28.30, *p* = .000, V = .310
-Difficult to extremely difficult	38 (21.0)	24 (31.6)	18 (51.4) ^c^		42 (31.8) ^c^	37 (50.0)	60 (68.2) ^c^	

^a^ Cumulative Lifetime Violence Severity

^b^ Unless otherwise specified

^c^ Standardized Residual > ± 1.96

### Gender

In the total sample, three subscales of the CMNI-46 were significantly associated (ES = small) with patterns of cannabis use: Playboy, Self-reliance, and Heterosexual Self-presentation (See Table 3). The mean scores on the Playboy subscale for *daily* cannabis and *sometimes* use were each significantly greater than those for *never* use suggesting that *daily* and *sometimes* use may be associated with endorsement of the norm of sexual activity with casual partners. For Self-reliance, those who reported *daily* use had significantly higher mean scores than those who reported *never* using cannabis. For Heterosexual Self-presentation, the mean scores for *daily* and *never* use were each significantly greater than for *sometimes* use*.*

**Table 3:** Patterns of cannabis use in past year by conformity to masculine norms (*N* = 589)

	Patterns of Cannabis Use (N = 589)			ANOVA
	Never (53.3%, *n =* 314) ^a^	Sometimes (once or twice, monthly or weekly) (25.6%, *n* = 151) ^a^	Daily or Almost Daily (21%, *n* = 124) ^a^	
CMNI^b^ Playboy Subscale: μ ± S.D.	1.06 ± 0.67^c d^	1.31 ± 0.66^c^	1.24 ± 0.64^d^	*F* (2, 586) = 8.09, *p* = .000, *η*^2^ = .027
CMNI^b^ Self-reliance Subscale: μ ± S.D.	1.35 ± 0.58^c^	1.49 ± 0.67	1.58 ± 0.52 ^c^	*F* (2, 284.41) = 8.25, *p* = .001, *η*^2^ = .024
CMNI^b^ Heterosexual Self-presentation Subscale: μ ± S.D.	1.26 ± 0.68^c^	1.05 ± 0.62^c d^	1.29 ± 0.78^d^	*F* (2, 276.65) = 6.039, *p* = .005, *η*^2^ = .018

^a^ Unless otherwise specified

^b^ Conformity to Masculine Norms Inventory

^c d^ Indicates significant differences between group mean scores using Games-Howell Post Hoc Tests

Patterns of use by gender norms differed in the lower and higher CLVS groups (see Table 4). In the lower group, the mean score on the Power over Women subscale for *never* use was significantly higher than that for *sometimes* use (ES = small). In contrast, patterns of cannabis use for the higher CLVS group were significantly associated with three subscales, similar to those for the total sample. For Playboy, the mean score for *sometimes* use was significantly greater than for *neve*r use. For Self-reliance, the mean scores for *daily* and *sometimes* cannabis use were significantly greater than for *never* use. For Heterosexual Self-presentation, the mean scores for *daily* and *never* use were each significantly greater than for *sometimes* use.

**Table 4:** Patterns of cannabis use in past year by conformity to masculine norms by lower and higher CLVS^a^ scale score groups

	Lower CLVS (*n* = 294) Patterns of Cannabis Use				Higher CLVS (*n* = 295) Patterns of Cannabis Use			
	Never (61.9%, *n =* 182)^b^	Sometimes (26.2%, *n* = 77)^b^	Daily (11.9%, *n* = 35) ^b^	ANOVA	Never (44.7%, *n =* 132) ^b^	Sometimes (25.1%, *n* = 74) ^b^	Daily (30.2%, *n* = 89) ^b^	ANOVA
CMNI^c^ Power over Women Subscale: μ ± S.D.	0.70 ± 0.52^c^	0.52 ± 0.41^c^	0.69 ± 0.46	*F* (2, 291) = 3.86, *p = .022, η*^2^ = .026				
CMNI^c^ Playboy Subscale: μ ± S.D.					1.08 ± 0.69^d^	1.44 ± 0.68^d^	1.26 ± 0.67	*F* (2, 292) = 6.69, *p* = .001, *η*^2^ = .044
CMNI^c^ Self-reliance Subscale: μ ± S.D.					1.40 ± 0.69^d e^	1.72 ± 0.69^d^	1.61 ± 0.52^e^	*F* (2, 292) = 7.28, *p* = .001, *η*^2^ = .048
CMNI^b^ Heterosexual Self-presentation Subscale: μ ± S.D.					1.24 ± 0.66^d^	0.95 ± 0.66^d e^	1.33 ± 0.77^e^	*F* (2, 292) = 6.61, *p* = .002, *η*^2^ = .043

^a^ Cumulative Lifetime Violence Severity

^b^ Unless otherwise specified

^c^ Conformity to Masculine Norms Inventory

^d e^ Indicates significant differences between group mean scores using Games-Howell Post Hoc Tests

### Health

In the total sample (see Table 5), *daily* cannabis use was significantly associated with high disability chronic pain (ES = small), and with possible depression, PTSD, and moderate or severe anxiety (ES = medium). *Daily* cannabis users also had a significantly higher mean number of chronic health problems than *sometimes* or *never* users (ES = small). Men who had *never* used cannabis in the past year were less likely to have possible depression, PTSD or anxiety (ES = medium).

**Table 5:** Patterns of cannabis use in past year by health indicators for total sample (*N* = 589)

	Patterns of Cannabis Use (N = 589)			Test Statistic
	Never (53.3%, *n =* 314) ^a^	Sometimes (once or twice, monthly or weekly) (25.6%, *n* = 151) ^a^	Daily or Almost Daily (21%, *n* = 124) ^a^	
Chronic Pain Disability: *n* (%)	(*n* = 313)			
-Low (CPG 0, 1, 2)	272 (86.9)	129 (85.4)	88 (71.0)	*χ*^*2*^ (2) = 16.85, *p* = .000, V = .169
-High (CPG 3, 4)	41 (13.1)	22 (14.6)	36 (29.0)^d^	
CESD-R Depression Indicator: n (%)				
-Yes (> 15)	75 (23.9) ^d^	62 (41.1)	73 (58.9) ^d^	*χ*^*2*^ (2) = 50.01, *p* = .000, V = .291
-No (< 16)	239 (76.1)^d^	89 (58.9)	51 (41.1) ^d^	
PCL-C PTSD Indicator: *n* (%)	(*n* = 313)			
-Yes (≥ 35)	69 (22.0) ^d^	59 (39.1)	70 (56.5) ^d^	*χ*^*2*^ (2) = 49.73, *p* = .000, V = .291
-No (< 35)	244 (78.0) ^d^	92 (60.9)	54 (43.5) ^d^	
GAD-7 ≥ 10 Moderate to Severe Anxiety Indicator: *n* (%)	(*n* = 313)			
-Yes (≥10)	38 (12.1) ^d^	37 (24.5)	40 (32.3) ^d^	*χ*^*2*^ (2) = 26.01, *p* = .000, V = .210
-No (< 10)	275 (87.9)	114 (75.5)	84 (67.7)	
Number of Chronic Health Problems Diagnosed by Health Care Provider ever: μ ± S.D.	1.86 ± 2.11^b^	1.84 ± 1.85^c^	2.88 ± 2.40^b c^	*F* (2, 279.23) = 9.55, *p* = .000, *η*^2^ = .038

^a^ Unless otherwise specified

^b c^ Indicates significant differences between group mean scores using Games-Howell Post Hoc Tests

^d^ Standardized Residual > ± 1.96

Patterns of cannabis use by health differed in the lower and higher CLVS groups (see Table 6). No significant associations were found for *sometimes* use in either group. In the lower CLVS group, *daily* cannabis users were significantly more likely to have high disability chronic pain, possible depression, PTSD, and moderate to severe anxiety, all with medium ES. *Never* users were significantly less likely to have possible PTSD. As well, no differences in patterns of cannabis use by number of chronic health problems were found. In the higher CLVS group no significant differences by pattern of cannabis use were found for chronic pain disability, or for number of chronic health problems according to post-hoc testing. *Daily* cannabis use was significantly associated with possible depression (ES = medium). Men who *never* used cannabis were significantly less likely to have possible depression or PTSD (ES = medium). Although patterns of use by anxiety were statistically significant overall in the higher CLVS group, the ES was small and no cells had significantly greater or smaller than expected counts based on standardized residuals.

**Table 6:** Patterns of cannabis use in past year by health indicators by lower and higher CLVS^a^ scale score groups

	Lower CLVS (*n* = 294) Patterns of Cannabis Use				Higher CLVS (*n* = 295) Patterns of Cannabis Use			
	Never (61.9%, *n =* 182)^b^	Sometimes (26.2%, *n* = 77)^b^	Daily (11.9%, *n* = 35) ^b^	Test Statistic	Never (44.7%, *n =* 132) ^b^	Sometimes (25.1%, *n* = 74) ^b^	Daily (30.2%, *n* = 89) ^b^	Test Statistic
Chronic Pain Disability: *n* (%)								
-Low (CPG 0, 1, 2)	167 (92.3))	73 (94.8)	26 (74.3)	*χ*^*2*^ (2) = 13.35, *p = .001*, V = .213	105 (79.5)	56 (75.7)	62 (69.7)	*χ*^*2*^ (2) = 2.81, *p* = .245, V = .098
-High (CPG 3, 4)	14 (7.7)	4 (5.2)	9 (25.7)^d^		27 (20.5)	18 (24.3)	27 (30.3)	
CESD-R Depression Indicator: *n* (%)								
-Yes (> 15)	28 (15.4)	19 (24.7)	15 (42.9) ^d^	*χ*^*2*^ (2) = 14.12,*p = .001*, V = .219	47 (35.6) ^d^	43 (58.1)	58 (65.2) ^d^	*χ*^*2*^ (2) = 21.07, *p =* .000, V = .267
-No (< 16)	154 (84.6)	58 (75.3)	20 (57.1)		85 (64.4) ^d^	31 (41.9)	31 (34.8) ^d^	
PCL-C PTSD Indicator: *n* (%)					(*n =* 131)			
-Yes (≥ 35)	18 (9.9) ^d^	14 (18.2)	14 (40.0) ^d^	*χ*^*2*^ (2) = 20.67, *p = .000*, V = .265	51 (38.9) ^d^	45 (60.8)	56 (62.9)	*χ*^*2*^ (2) = 15.50, *p = .000*, V = .230
-No (< 35)	164 (90.1)	63 (81.8)	21 (60.0)		80 (61.1) ^d^	29 (39.2)	33 (37.1)	
GAD-7 ≥ 10 Moderate to Severe Anxiety Indicator: *n* (%)					(*n =* 131)			
-Yes (≥10)	9 (4.9)	8 (10.4)	8 (22.9) ^d^	*χ*^*2*^ (2) = 12.58, *p = .002*, V = .207	29 (22.1)	29 (39.2)	32 (36.0)	*χ*^*2*^ (2) = 8.19, *p = .017*, V = .167
-No (< 10)	173 (95.1)	69 (89.6)	27 (71.1)		102 (77.9)	45 (60.8)	57 (64.0)	
Number of Chronic Health Problems Diagnosed by Health Care Provider ever: μ ± S.D.	1.40 ± 1.70	1.30 ± 1.67	2.11 ± 2.25	*F* (2, 81.19) = 1.87, *p = .062, η*^2^ = .019	2.48 ± 2.44	2.41 ± 1.86	3.18 ± 2.41	*F* (2, 294) = 3.11, *p = .046*^*c*^*, η*^2^ = .021

^a^ Cumulative Lifetime Violence Severity

^b^ Unless otherwise specified

^c^ No significant differences found between groups with Games-Howell post-hoc testing

^d^ Standardized Residual > ± 1.96

### Substance use

Patterns of cannabis use varied significantly by other substance use in the total sample (see Table 7). *Daily* use was significantly associated with currently smoking cigarettes (ES = large), use of prescription drugs for non-medical reasons or in more than prescribed amounts in the past year (ES = medium), and use of street/recreational drugs in the past year (ES = large). *Sometimes* use was significantly associated with possible Alcohol Use Disorder and binge drinking (ES = medium). *Never* users were significantly less likely to be current smokers, possible hazardous drinkers, binge drinkers, and users of street drugs or prescription drugs for non-medical purposes.

**Table 7:** Patterns of cannabis use in past year by substance use for the total sample (*N* = 589)

	Patterns of Cannabis Use			Chi-square
	Never (53.3%, *n =* 314) ^a^	Sometimes (once or twice, monthly or weekly) (25.6%, *n* = 151) ^a^	Daily or Almost Daily (21%, *n* = 124) ^a^	
Smoking: *n* (%)	(*n* = 313)			
-Current Smoker	36 (11.5) ^d^	43 (28.5)	65 (52.4) ^d^	*χ*^*2*^ (4) = 107.47, *p = .000,* V = .302
-Quit Smoking	72 (23.0)	41 (27.2)	38 (30.6)	
-Never Smoked	205 (65.5) ^d^	67 (44.4)	21 (16.9) ^d^	
AUDIT-C Indicator of Possible Hazardous Drinking or Active Alcohol Use Disorder: *n* (%)	(*n* = 313)			
-Yes	122 (39.0) ^d^	104 (68.9) ^d^	75 (60.5)	*χ*^*2*^ (2) = 41.90, *p = .000,* V = .267
-No	191 (61.0) ^d^	47 (31.1) ^d^	49 (39.5)	
Binge Drinking (6 or more drinks monthly or more often): *n* (%)				
-Yes	69 (22.0) ^d^	77 (51.0) ^d^	46 (37.1)	*χ*^*2*^ (2) = 40.53, *p = .000,* V = .262
-No	245 (78.0) ^d^	74 (49.0) ^d^	78 (62.9)	
Use of prescription drugs^b^ for non-medical reasons or in more than prescribed amounts in past year: *n* (%)				
-Yes	21 (6.7) ^d^	28 (18.5)	41 (33.1) ^d^	*χ*^*2*^ (2) = 49.45, *p = .000,* V = .290
-No	293 (93.3)	123 (81.5)	83 (66.9) ^d^	
Use of street/recreational drugs^c^ other than cannabis in past year: *n* (%)		(*n* = 150)		
-Yes	5 (1.6) ^d^	30 (20.0)	56 (45.2 ^d^	*χ*^*2*^ (2) = 132.15, *p = .000,* V = .474
-No	309 (98.4) ^d^	120 (80.0)	68 (54.8) ^d^	

^a^ Unless otherwise specified

^b^ such as codeine, morphine, dilaudid, fentanyl, oxycodone or valium

^c^ such as, cocaine, crack, heroin, opium, speed, crystal meth, LSD, acid, mushrooms, ecstasy, special K

^d^ Standardized Residual > ± 1.96

Patterns of cannabis and other substance use were significantly associated in both CLVS groups (see Table 8). In both groups, *daily* cannabis use was associated with smoking cigarettes currently (ES = large), and using street drugs (ES = large) and prescription drugs for non-medical reasons (ES = medium). *Sometimes* users were more likely to be hazardous and binge drinkers in both CLVS groups (ES = medium). Only in the lower group was *sometimes* use associated with using street drugs. In the lower CLVS group, *never* users of cannabis were more likely to have never smoked, and less likely to have engaged in binge drinking, and use of prescription and street drugs. In the higher CLVS group, *never* users were less likely to be current smokers, hazardous drinkers, binge drinkers, and users of prescription or street drugs and more likely to have never smoked.

**Table 8:** Patterns of cannabis use in past year by substance use by by lower and higher CLVS^a^ scale score groups

	Lower CLVS (*n* = 294) Pattern of Cannabis Use				Higher CLVS (*n* = 295) Pattern of Cannabis Use			
	Never (61.9%, *n =* 182)^b^	Sometimes (26.2%, *n* = 77)^b^	Daily (11.9%, *n* = 35) ^b^	Chi- square	Never (44.7%, *n =* 132) ^b^	Sometimes (25.1%, *n* = 74) ^b^	Daily (30.2%, *n* = 89) ^b^	Chi- square
Smoking: *n* (%)	(*n* = 181)							
-Current Smoker	22 (12.2)	15 (19.5)	14 (40.0) ^e^	*χ*^*2*^ (4) = 35.29, *p = .000,* V = .245	14 (10.6) ^e^	28 (37.8)	51 (57.3) ^e^	*χ*^*2*^ (4) = 63.32, *p = .000*, V = .328
-Quit Smoking	29 (16.0)	23 (29.9)	13 (37.1)		43 (32.6)	18 (24.3)	25 (28.1)	
-Never Smoked	130 (71.8) ^e^	39 (50.6)	8 (22.9) ^e^		75 (56.8) ^e^	28 (37.8)	13 (14.6) ^e^	
AUDIT-C Indicator of Possible Hazardous Drinking or Active Alcohol Use Disorder: *n* (%)					(*n =* 131)			
-Yes	75 (41.2)	52 (67.5) ^e^	19 (54.3)	*χ*^*2*^ (2) = 15.33,*p = .000,* V = .228	47 (35.9) ^e^	52 (70.3)^e^	56 (62.9)	*χ*^*2*^ (2) = 27.77, *p = .000,* V = .307
-No	107 (58.8)	25 (32.5) ^e^	16 (45.7)		84 (64.1) ^e^	22 (29.7) ^e^	33 (37.1)	
Binge Drinking (6 or more drinks monthly or more often): *n* (%)								
-Yes	44 (24.2) ^e^	42 (54.5) ^e^	16 (45.7)	*χ*^*2*^ (2) = 24.15, *p = .000,* V = .287	25 (18.9) ^e^	35 (47.3) ^e^	30 (33.7)	*χ*^*2*^ (2) = 18.60, *p = .000,* V = .251
-No	138 (78.0)	35 (45.5) ^e^	19 (54.3)		107 (81.1)	39 (52.7)	59 (66.3)	
Use of prescription drugs^c^ for non-medical reasons or in more than prescribed amounts in past year: *n* (%)								
-Yes	12 (6.6) ^e^	12 (15.6)	11 (31.4) ^e^	*χ*^*2*^ (2) = 18.61, *p = .000,* V = .252	9 (6.8) ^e^	16 (21.6)	30 (33.7) ^e^	*χ*^*2*^ (2) = 27.06, *p = .000,* V = .296
-No	170 (93.4)	65 (84.4)	24 (68.6)		123 (93.2)	58 (78.4)	59 (66.3)	
Use of street/recreational drugs^d^ other than cannabis in past year: *n* (%)						(*n* = 73)		
-Yes	1 (0.5) ^e^	15 (19.5) ^e^	16 (45.7) ^e^	*χ*^*2*^ (2) = 69.68, *p = .000, V = .487*	4 (3.0) ^e^	15 (20.5)	40 (44.9) ^e^	*χ*^*2*^ (2) = 58.23, *p = .000,* V = .445
-No	181 (99.5)	62 (80.5)	19 (54.3) ^e^		128 (97.0) ^e^	58 (79.5)	49 (55.1) ^e^	

^a^ Cumulative LifetimeViolence Severity

^b^ Unless otherwise specified

^c^ such as codeine, morphine, dilaudid, fentanyl, oxycodone or valium

^d^ such as, cocaine, crack, heroin, opium, speed, crystal meth, LSD, acid, mushrooms, ecstasy, special K

^e^ Standardized Residual > ± 1.96

## Discussion

These findings make several contributions to our understanding of the patterns of cannabis use prior to non-medical use legalization among a community sample of Canadian men, specifically those living in the eastern province of NB. Notably, the overall prevalence of cannabis use in the *MVGHS* sample in the past year (46.7%) was almost twice that reported in the 2018 Canadian Cannabis Survey (CCS) for NB men (26.1%) and for Canadian men (26.5%) (Health Canada 2018). Some of this difference may be accounted for by age; men in the 2018 CCS were aged 16 years and older as compared to 19 to 65 years in the *MVGHS*. Interestingly, the 2017 U.S. National Survey on Drug Use and Health (NSDUH) rate of cannabis use in the past year for men aged 12 and older was also much lower at 17.8% (Center for Behavioral Health Statistics and Quality 2018).

Unlike cannabis research that relies on prevalence rates derived from a dichotomous measure (i.e., yes/no) of cannabis use in a specific period (e.g., past 12 months or past 3 months), we analysed data on a pattern of use categorized as *daily*, *sometimes*, or *never*. Association between health and social indicators and pattern of use is important because the frequency *daily or almost daily* is applied in epidemiological studies as a proxy for heaviest use, identifying those who may be at greater risk (World Health Organization 2016). Of the 275 men in the *MVGHS* who had used cannabis in the past year, 45.1% (*n* = 124) reported using *daily*, a rate almost twice that of the 27.9% of Canadian men who used cannabis *daily* or almost daily in the past year reported in the 2018 CCS (Health Canada 2018). Therefore, the proportion of NB men at possible risk for problematic cannabis use may be considerably higher than for Canadian men in general.

Despite violence exposure not being a criterion for taking part in this study, 97.5% of participants reported experiences of violence on the CLVS scale. This high prevalence is consistent with violence being a major public health problem for men (Haegerich and Hall 2011) and may be attributed to the CLVS scale measuring a comprehensive range of violence experiences as target and/or perpetrator across the lifespan (Scott-Storey et al. 2018). Nonetheless, applicability of findings may be limited to men living primarily in widely dispersed medium-sized cities, towns, and rural areas similar to the province of NB. Our findings add to our understanding of patterns of cannabis use and lifetime cumulative violence. In a previous analysis, we dichotomized our measure of cannabis use as *daily or almost daily* and *not daily* (i.e., never, once or twice, monthly, weekly) and found that men in the higher CLVS group were significantly more likely to use *daily* and less likely to use *not daily* than men with lower CLVS (Scott-Storey et al. 2018). Our current analysis supports our past finding by showing that men with higher CLVS were more likely to use cannabis *daily* as compared to men with lower CLVS (ES = medium) but expands our knowledge of the relationships among *never* and *sometimes use* and lifetime cumulative violence. Specifically, men in the higher CLVS group were significantly less likely to use *never* but neither more or less likely to use *sometimes* than men in the lower CLVS group, suggesting that lifetime cumulative violence may not be a factor in occasional cannabis use, but may be for daily use. Although our analysis has expanded our understanding of these relationships, further study of cannabis use patterns and other sociodemographic variables in CLVS groups is needed.

In both the total sample and the higher CLVS group, significantly more men than expected ages 19 to 24 years had used cannabis *sometimes* in the past year and the number of *daily* users did not differ from expected (ES = medium). This may signify that more younger men, particularly those with histories of higher CLVS, are choosing to use cannabis occasionally rather than daily, a consumption pattern consistent with guidelines recommended by Fischer et al. (2017) to prevent the health and social consequences of intensive use. In addition, we found that significantly more men than expected in the total sample and the higher CLVS group who were single, divorced or separated used cannabis both *sometimes* and *daily* whereas significantly fewer married or cohabiting men used *sometimes* or *daily* (ES = large)*.* Although lower prevalence of cannabis use among married men has been reported elsewhere (Han et al. 2017; Merline et al. 2004), dichotomous measurement of cannabis use and non-use in these studies does not permit differentiation between *daily* and *sometimes* use and their unique associations with marital status. Our findings highlight the importance of using non-dichotomous measures of cannabis in analysis to enable exploration of meaningful patterns of usage in the context of lifetime cumulative violence over time for the purpose of identifying who may be at lesser or greater risk for cannabis-related problems.

In the *MVGHS* total sample, *daily* cannabis use was associated with having a high school education or less, being unemployed, having an annual income of less than $25,000 CAD and having difficulty living on one’s income, with medium ES. With respect to education and difficulty living on income, this pattern was also evident in both CLVS groups, suggesting that irrespective of lifetime cumulative violence exposure, these factors are associated with greater *daily* cannabis use. In contrast, the significant and medium relationship between *daily* cannabis use and unemployment in the total sample held for the higher CLVS group but not for the lower CLVS group, suggesting that severity of lifetime cumulative violence influences this association.

Our socio-demographic findings are in line with data from the 2018 CCS which showed that men with the highest prevalence of cannabis use in the previous 12 months were those who had a high school diploma (34.9%) or less (29.0%), were unemployed (34.4%), and had an income under 10,000 CAD (35.2%) or from 10,000 to 24,999 CAD (37.5%) (Health Canada 2018). Because overall unemployment rates in NB were 8.1% in 2017, higher than the national Canadian rate of 6.5% (NBjobs 2018), NB men may be at greater risk for *daily* cannabis use than their national counterparts. Other research suggests that unemployment in early adulthood is associated with concurrent and ongoing cannabis use through mid-life, and that early adulthood use of cannabis is associated with decreased subsequent participation in the workforce (Hara et al. 2013). Thus, the 13.5% unemployment rate for NB youth aged 15 to 24 years (NBjobs 2018) could have implications not only for the prevalence of *daily* cannabis use but also for the participation of NB men in the workforce into middle adulthood. A preventive strategy for reducing *daily* cannabis use among NB men might be strengthening employment opportunities for youth. Overall, our findings lend support to health promotion strategies targeted to socio-demographic factors associated with daily cannabis use.

Our efforts to address the knowledge gap regarding how gender, that is ideas about what it means to be a man, influences patterns of cannabis use are important because, in Canada, men have been found to be significantly more likely than women to both try cannabis and to exhibit patterns of cannabis use deemed problematic (Bonner et al. 2017). Although total scores of multidimensional gender measures reflecting greater adherence to dominant masculine norms have, for the most part, been associated with detrimental health outcomes such as substance use, associations between unidimensional subscale scores and health outcomes are often mixed, with some found to be protective (Gerdes and Levant 2018). Such findings highlight the complex relationships between gender and health outcomes that may be culturally, situationally or contextually dependent (Gerdes and Levant 2018). Our analysis of relationships between CMNI-46 subscales and patterns of cannabis use among NB men ages 19 to 65 years was additionally nuanced because our measure of cannabis use was not dichotomous (i.e., yes/no). Using a dichotomous cannabis use measure, Liu and Iwamoto (2007) in a sample of Asian-American university students, found cannabis use was associated with the norm of Playboy, a result consistent with our finding for the total sample that *daily* and *sometimes* users were more likely than *never* users to endorse having sexual activity with casual partners as a masculine norm. However, our findings for the norm of Heterosexual Self-presentation for the total sample suggests a more complex relationship than their finding of cannabis use being associated with this norm (Liu and Iwamoto 2007). We found that *sometimes* users had fewer concerns about how others perceive their sexual orientation than *never* or *dail*y users, suggesting that *never* and *daily* users, despite their distinctly different usage patterns, adhere more strongly to the dominant masculine norm of Heterosexual Self-presentation than occasional users. The cultural dissimilarity of the two samples may account for this difference and also for our finding that *daily* use was associated with the norm of Self-reliance in the total sample, an association not found in the Asian American sample. Overall, although the relationships for each gender indicator and patterns of cannabis use were significant, because the ES was small our findings for the total sample must be interpreted with caution.

Our findings further suggest that lifetime cumulative violence may be a contextual factor that influences how particular masculine gender norms relate to patterns of cannabis use. In the higher CLVS group, significant relationships were found between the pattern of cannabis use and the same gender norms as in the total sample. Although the patterns of use for Heterosexual Self-presentation were the same in the higher CLVS group as those for the total sample, they differed for Self-reliance and Playboy. Both *daily* and *sometimes* users had significantly higher scores on self-reliance than *never* users, suggesting that men with higher CLVS scores may value the belief that men should solve their own problems and avoid seeking help. It may be that men with higher CLVS had historically been unable to count on others. With respect to Playboy, only *sometimes* users had significantly higher endorsement scores compared to *never* users, an outcome that is difficult to interpret. For the lower CLVS group, the finding that *never* users had significantly higher scores on the norm of having control over women than *sometimes* users is also puzzling. These discoveries, particularly those for the higher CLVS group, highlight the need for further research examining the contextual intersections between gender, CLVS and patterns of cannabis use.

One limitation in the *MVGHS* is our failure to collect data about whether men’s intent in using cannabis was for recreational and/or therapeutic purposes. Prior to legalization of cannabis for non-medical use, Canadians engaged in therapeutic use either as authorized users of approved medical cannabis or as unauthorized therapeutic users of recreational sources; however, few differences were found between these groups with respect to medical conditions and patterns of cannabis use (Walsh et al. 2013). In interpreting our finding of patterns of cannabis use by health problem, we recognize that cannabis use may not be intentionally therapeutic. Nonetheless, our finding for the full sample that men who used cannabis *daily* had significantly more chronic illnesses (ES = small) than men who used *sometimes* or *never* suggests it is possible that they may be using for therapeutic purposes. Further, the fact that no significant differences were found by pattern of cannabis use and in either the lower or higher CLVS group, suggests that lifetime cumulative violence severity may not influence usage related to number of chronic health problems.

Chronic pain has been found to be one of the main reasons for therapeutic use of cannabis with regular users of all ages attempting to manage their chronic pain with cannabis (Walsh et al. 2013; Fales et al. 2019). In the total sample, 16.8% (*n* = 99) of men were found to have high disability chronic pain, a rate similar to the 18.9% overall rate for Canadians over 18 years of age who were assessed with persistent, repeated, recent, intense chronic pain (Schopflocher et al. 2011). Our finding (ES = small) that significantly more of the *MVGHS* men with high disability chronic pain than expected used cannabis used *daily* (36.4%, *n* = 36) and neither more nor less than expected used *sometimes* (22.2%, *n* = 22), suggests that more than a third might be using cannabis therapeutically. Given the established relationship between violence and chronic pain (Hart-Johnson and Green 2012), we cannot explain why there were no significant differences in patterns of cannabis use by pain disability in the higher CLVS group whereas in the lower CLVS group significantly more men than expected with higher disability pain used cannabis *daily* (ES = medium). Notably, 72.7% (*n* = 72*)* of the men with high disability chronic pain were in the higher CLVS group.

The recent legalization of cannabis for non-medical use in Canada has kindled increased interest in understanding patterns of cannabis use and mental health. Our finding that men in the total sample with possible depression were significantly more likely to use cannabis *daily* and less likely to *never* use than men without possible depression (ES = medium) is consistent with outcomes of many cross-sectional studies (Volkow et al. 2014). Crucially, the credibility of these findings is limited by failure to consider other factors that when controlled often eliminate the significant relationship between cannabis use and depression (WHO 2016; Volkow et al. 2014). For example, in a 3-year Swedish study of men and women ages 20 to 64 years, cannabis use no longer predicted possible depression when gender and serious family tensions were controlled, suggesting that these social determinants, not cannabis, might account for the depressive symptoms (Danielsson et al. 2016). Our finding that significantly more men than expected in both the lower and higher CLVS groups (both with medium ES) who had possible depression used cannabis *daily* must be interpreted cautiously. Although it implies that lifetime cumulative violence severity may not influence the cannabis-depression association, further study of this relationship is needed because the frequency of depression in the higher CLVS group of 50.2% (*n* = 148) of which 39.2% (*n* = 58) used cannabis *daily* is much higher than in the lower CLVS group at 21.1% (*n* = 62) of which 24.2% (*n* = 15) used *daily*.

In our total sample, significantly more men than expected who met the criteria for possible PTSD used cannabis *daily* and fewer used cannabis *never* (ES = medium)*.* Of the 210 men with possible PTSD, 61.4% (*n* = 129) reported using cannabis in the past year, 70 *daily* and 59 *sometimes*, suggesting that some men may use cannabis to cope with symptoms of PTSD, a strategy found to be potentially useful in a recent Canadian systematic review of medical cannabis use and mental health (Walsh et al. 2017). Similar to depression, fewer men in the lower CLVS group (15.6%; *n* = 46) had possible PTSD than those in the higher CLVS group (51.7%, *n* = 152), a finding consistent with violence being an important factor in the development of PTSD (American Psychiatric Association 2013). Although our findings were significant for possible PTSD in the in the higher CLVS group (ES = medium), cell counts were only different than expected for *never* users. Although counts for *daily* (*n* = 56) and *sometimes* (*n* = 45) use did not differ from expected, it is notable that 66.4% (*n* = 101) of men with higher CLVS and possible PTSD had been using cannabis in the past year. Similar to PTSD, possible moderate to severe anxiety in the total sample was significantly associated with more men than expected using cannabis *daily*, and fewer using *never* (ES medium), a finding that is not surprising given that relief of anxiety is one of the most widely reported reasons for therapeutic cannabis use (Walsh et al. 2017). Despite such reports, a recent review indicated that robust evidence supporting the therapeutic effects of cannabis on anxiety is lacking and that longitudinal prospective studies and clinical trials are needed to determine its usefulness (Lowe et al. 2019). It is difficult to interpret our findings that a) in the lower CLVS group significantly more men than expected with anxiety used cannabis *daily* (ES medium), and b) in the higher CLVS group, despite a small ES and a statistically significant association between anxiety and patterns of cannabis use, no significant differences in cell counts were found. Further study is required to unravel the relationships among lifetime cumulative violence, patterns of cannabis use and possible anxiety.

Our findings of significant relationships for use of other substances and patterns of cannabis use in the total sample and both CLVS groups contribute to the developing knowledge base. Significantly more *daily* users (52.4%) than expected in the total sample were also current cigarette smokers, a prevalence rate similar to the 55.5% of men in a US NSDUH survey (Pacek et al. 2018). Additionally, 30.6% of *daily* users in the MVGHS had quit smoking, a finding that may be explained by a general decline in NB in cigarette smoking from 1998 to 2017 (Reid et al. 2019). Similar patterns of cigarette use were found in the lower and higher CLVS groups, although a smaller proportion of daily cannabis users were current smokers in the lower CLVS group as compared to the higher CLVS group. Cigarette smoking has been found to be a coping mechanism more likely to be employed by those experiencing greater violence severity, particularly intimate partner violence (Crane et al. 2013).

Our findings related to cannabis and alcohol use suggest the need for further research in this area. Among men who had used cannabis in the past year, 65.1% (*n* = 179) met the criteria for possible hazardous drinking, a rate slightly higher than that of 57.3% of cannabis users found in the 2017 Canadian Tobacco, Alcohol and Drugs Survey (CTADS) to be heavy drinkers (Statistics Canada 2018). In the total sample and both CLVS groups, we found a significant association between patterns of cannabis use and possible hazardous drinking (ES = medium), specifically that more *sometimes* users and fewer *never* users than expected were possible hazardous drinkers; the number of *daily* users was not significantly different than expected. Comparable significant associations and ES were found for binge drinking. This finding is important because it suggests that NB men who use cannabis occasionally may be most at risk for being possible hazardous drinkers and/or binge drinkers as compared to those who use daily or never. Similarly, in a large study of college students in France, the adjusted odds of being a frequent binge drinker were increased almost 5-fold for being male and 13-fold for using cannabis occasionally (Tavolacci et al. 2016). Another important implication of our findings is that lifetime cumulative violence does not appear to affect the relationship between patterns of cannabis use and either hazardous or binge drinking. A limitation of these study findings is that we do not know whether men in the sample used cannabis and alcohol simultaneously (i.e., together at the same time to complement one another) or concurrently (i.e., one at a time as a substitute for the other). Based on United States National Alcohol Survey findings, the prevalence of simultaneous alcohol and cannabis use was almost twice that of concurrent use and associated with increased frequency and quantity of alcohol consumption (Subbaraman and Kerr 2015). A future inquiry examining simultaneous versus concurrent use might yield critical information for identifying men at risk.

Of men in the total sample who used cannabis in the past year, 86 (31.3%) had used street drugs, a rate somewhat higher than the 23.8% of male cannabis users over age 15 in the 2017 CTADS who reported using illegal drugs (Statistics Canada 2018). It is unclear how illegal drugs are defined by CTADS and it is possible that prescription drugs used for non-medical purposes are included in this category. In our sample*,* 69 (25.1%) of cannabis users also reported using prescription drugs for non-prescription purposes. Significantly more *daily* cannabis users than expected were found to use street drugs (ES = large) and also to use prescription drugs for non-medical purposes (ES = medium) in the total sample and both CLVS groups. Together these findings suggest consistency of association between patterns of cannabis use and that of other substances that is not affected by lifetime cumulative violence severity in this sample of men.

## Conclusion

These analyses begin to fill a gap in knowledge about patterns and correlates of cannabis use necessary to inform health promotion initiatives in the wake of cannabis legalization for non-medical use. Our analysis reflects usage among a group often poorly represented in larger cannabis surveys; that is, eastern Canadian men who live in small to medium-sized cities, towns and rural areas of a sparsely populated province. Given that our data were collected prior to legalization of cannabis, our findings of prevalence of use and daily usage in the past year approaching twice that of the Canadian population is remarkable and signals the urgent need for further study of cannabis use among such sub-groups of men. Two limitations of our study that must be addressed in future research are: a) lack of data regarding the dosage of cannabis used (Asbridge et al. 2014), and b) the categorical measure of cannabis use that limits the range of possible multivariate statistical approaches for analysis. Nonetheless, an important contribution of this work is the demonstrated usefulness of a non-dichotomous measure of cannabis use that captures *sometimes* as well as *daily* use. In particular, the relationship found between *sometimes* use and possible hazardous drinking is noteworthy and necessitates further inquiry. Our results related to health outcomes add support to the existing call for future longitudinal studies that control for potential confounding variables to disentangle the nature (e.g., cause, consequence, mediator) of relationships among cannabis use, socio-demographic characteristics and health, particularly in the context of lifetime cumulative violence. Further, our bivariate findings demonstrating statistical significance and strength of association between patterns of cannabis use and socio-demographic, gender, and health indicators will inform the construction of theoretical models for testing these complex relationships via future research.

Our findings add substantively to our previous finding that cannabis use was more likely among men with higher CLVS (Scott-Storey et al. 2018), by demonstrating how different patterns (*never, sometimes, daily*) of cannabis use are associated with socio-demographic, health and other substance use according to lifetime cumulative violence severity. Further, this is one of few studies where the relationships among patterns of cannabis use and men’s perceptions of what it means to be a man have been studied in the context of lifetime cumulative violence and our findings provide important groundwork for future research with this focus. Overall, our findings provide a beginning foundation for targeting community health promotion strategies regarding cannabis use.

## Data Availability

The datasets used and analysed during the current study are available from the corresponding author on reasonable request.

## Abbreviations

ANOVA: Analysis of variance
AUDIT-C: Audit Alcohol Consumption screen
CAD: Canadian dollar
CCS: Canadian Cannabis Survey
CESD-R: Center for Epidemiologic Studies-Depression Revised
CLVS: Cumulative Lifetime Violence Severity
CMNI-46: 46 item Conformity to Masculine Norms Inventory
CPG: Chronic Pain Grade
CTADS: Canadian Tobacco, Alcohol and Drugs Survey
ES: Effect size
GAD-7: Generalized Anxiety Disorder 7
MVGHS: Men’s Violence, Gender and Health Study
NB: New Brunswick
NSDUH: National Survey on Drug Use and Health
PCL-C: Posttraumatic Stress Disorder Checklist, Civilian Version
PTSD: Posttraumatic Stress Disorder
